# The contemporary epidemiology, causes, and management of drug-induced liver injury

**DOI:** 10.1097/HC9.0000000000001011

**Published:** 2026-07-20

**Authors:** Linea Swanson, Robert J. Fontana

**Affiliations:** Division of Gastroenterology and Hepatology, University of Michigan Medical School, Ann Arbor, Michigan, USA

**Keywords:** acute liver failure, causality assessment, drug hepatotoxicity, genetic polymorphisms, liver transplantation, RECAM

## Abstract

Drug-induced liver injury is an important cause of clinically significant liver disease. In Western countries, antibiotics are the leading cause of DILI, with amoxicillin–clavulanate and trimethoprim–sulfamethoxazole most frequently implicated. Herbal and dietary supplements (HDS) are increasingly implicated as a cause of DILI worldwide, including ashwagandha, turmeric, and green tea extract in the United States. Advances in DILI diagnosis include the development of the Revised Electronic Causality Assessment Method (RECAM) instrument and the creation of the LiverTox website with drug and HDS likelihood scores. Newer causes of DILI include kinase inhibitors for cancer and autoimmune conditions, various immunomodulatory agents, and medications to treat liver disease. Immune-mediated liver injury from checkpoint inhibitors (ILICI) typically presents within the first 6 months of treatment with asymptomatic laboratory abnormalities. Treatment of DILI remains primarily supportive by withholding the offending agent and close monitoring, with corticosteroids only showing benefit in highly selected patients. Over 80% of DILI patients will fully recover within 6 months of drug discontinuation with supportive care, but recent outcomes for DILI-related ALF due to HDS have been worsening. Prognostic models can help identify hospitalized DILI patients in need of transplant and others who are at increased risk for 6-month mortality. Whole-exome sequencing to identify new genetic risk factors, development of in vitro diagnostic assays, and further studies of the host adaptive immune response in DILI pathogenesis will hopefully lead to further improvements in DILI diagnosis and treatment.

## INTRODUCTION

Idiosyncratic drug-induced liver injury (DILI) is an infrequent but important cause of clinically significant acute and chronic liver injury. In addition to being uncommon with most marketed drugs (ie, <1 in 3000 exposed patients), the clinical phenotype and severity of DILI due to a specific agent can vary substantially. Host age, gender, and underlying diseases are not reliable risk factors, nor is the dose of the drug administered, but most cases arise within the first 6 months of drug initiation. Following drug discontinuation, the majority of patients with idiosyncratic DILI will improve, but up to 5%–10% may experience a fatal outcome, and 10%–20% may develop chronic liver injury. Multiple studies have demonstrated drug-specific human leukocyte antigen (HLA) associations with DILI susceptibility, implicating the adaptive immune response in its pathogenesis.

Over the past 20 years, the causes of idiosyncratic DILI in the United States have significantly evolved, with fewer cases attributed to antibiotics, anti-tuberculosis (TB) drugs, and aromatic anticonvulsants and an increasing proportion attributed to herbal and dietary supplements (HDS) and oncological agents. The aim of this paper is to review newly identified causes of DILI and to outline the currently recommended approach to the medical evaluation and management of DILI patients.

## EPIDEMIOLOGY

Determining the true incidence of idiosyncratic DILI is challenging, given the difficulty in confidently establishing a diagnosis. However, understanding the incidence and etiologies of DILI is vitally important for practitioners, regulatory authorities, and drug developers. Idiosyncratic DILI is responsible for <5% of acute liver injury cases evaluated by a hepatologist.^1^ A recent meta-analysis of 14 studies reported the pooled incidence of DILI to be 4.9 per 100,000 person-years, though significant variability was noted among countries.^2^ Prospective population-based studies from Iceland and France report an incidence of 19.1 and 13.9 cases per 100,000 persons, respectively.^3^^,^^4^ In comparison, population-based studies in Delaware and Sweden reported lower rates of DILI at 2 per 100,000 person-years.^5^^,^^6^ Retrospective data from mainland China suggest a higher incidence of DILI in hospitalized patients with an estimated annual incidence of 23.8 per 100,000 person-years.^7^ These data highlight the potential for significant under-reporting of DILI due to the lack of reliable diagnostic tests and billing codes for case identification.^4^

Several prospective DILI registries provide a detailed characterization of patient demographics, clinical characteristics, and trends in culprit medications and outcomes over time^8^^–^^13^ (Table 1). Patients enrolled in these studies were 49–59 years of age with a slight female predominance. Antimicrobials, including anti-TB drugs, were the most common culprit medications, with amoxicillin/clavulanate (AC) being the most frequently implicated individual agent in Western countries. The observed regional variations are likely due to differences in disease burden (particularly TB), physician prescribing habits, and medication and HDS availability in the population.

**TABLE 1:** Presenting features and outcomes with DILI reported in registry studies

	USA	Europe	Spanish	Latin America	India	Japan
Registry (ref)	DILIN^8^	Pro-Euro DILI registry^9^	Spain^10^	Latin America^11^	Indian Network of DILI^12^	Japanese DILI registry^13^
Years	2004–2013	2016–2021	1994–2018	2011–2022	2013–2018	2010–2018
Number of cases	899	246	843	483	1288	307
Median age (y)	49 (17)	56 (18)	54 (18)	49 (18)	43 (17)	59 (16)
Female %	59%	57%	48%	62%	49%	59%
Leading drug classes %	42% antimicrobials (AC, isoniazid, nitrofurantoin) 9.8% CV agents 9.1% CNS agents	37% antimicrobials (AC, flucloxacillin) 25% antineoplastic and immunomodulators 11% CV agents 6% HDS	40% antimicrobials (AC, anti-TB, fluoroquinolones) 12% CNS agents 11% CV agents 11% MSK agents	31% antimicrobials (AC, anti-TB) 12% MSK drugs 11% antineoplastic and immunomodulators	46% anti-TB 8.1% AED 6.5% other antimicrobial	11% antimicrobials 11% anti-inflammatory 10% antineoplastic
HDS (%)	16.1%	6%	3.4%	9%	13.9%	15%
Liver-related death (%)	3.0%	1.2%	2.1%	2.1%	6.1%	0%
Liver transplant (%)	4.0%	2.5%	1.5%	1.9%	0%	0%
Chronic DILI (%)	17%	NR	4.6%	12%	NR	NR

Abbreviations: AC, amoxicillin–clavulanate; AED, anti-epileptic drugs; CNS, central nervous system; CV, cardiovascular; DILI, drug-induced liver injury; DILIN, Drug-Induced Liver Injury Network; HDS, herbal and dietary supplements; MSK, musculoskeletal; NR, not reported; TB, tuberculosis; USA, United States of America.

Various herbal and botanical supplements have been used for centuries in Asian countries to treat a multitude of conditions and ailments, with increasing use also reported in Western countries. Nearly 60% of adult Americans reported taking an HDS product in the last 30 days between 2017 and 2018, with the frequency of HDS use greatest in older adults.^14^ Interestingly, the number of DILI cases due to HDS significantly increased from 7% to 20% between 2004 and 2012 in the Drug-Induced Liver Injury Network (DILIN).^15^ In addition, the proportion of acute liver failure (ALF) cases attributed to various HDS products climbed from 9.7% in 1998–2007 to 22% in 2008–2018 in the US Acute Liver Failure Study group.^16^ In the DILI registries, the proportion of cases attributed to an HDS product varied from 3.4% to 16.1% (Table 1). In Singapore, China, and Korea, HDS products have surpassed prescription medications as suspect agents.7,17,18 In Latin America, Camellia sinensis, Garcinia cambogia, and Herbalife products accounted for almost half of the HDS cases, while in DILIN, green tea extract, anabolic steroids, and turmeric-containing products were the leading causes.^11^^,^^19^

The incidence of DILI due to a specific drug is even more difficult to accurately establish. An analysis of DILIN data that was combined with population-based Medical Expenditure Panel Survey (MEPS) data was recently reported.^20^ Azithromycin had the highest estimated annual incidence of DILI at 1 case per 350 new prescriptions, older anti-epileptic drugs (AEDs) were one DILI case per 350–2500 prescriptions, and a subset of antibiotics [minocycline, AC, nitrofurantoin, terbinafine, trimethoprim–sulfamethoxazole (TMP/SMX)] had an estimated incidence of 1 DILI case per 1000–5000 prescriptions.^20^ In addition, among hospitalized patients in Iceland, AC was the most frequent drug associated with DILI (ie, 1 per 1300 patients), while TMP/SMX had the highest overall proportional risk at ~1 per 1000 new prescriptions.^21^

## DILI DIAGNOSIS AND NEW CAUSALITY ASSESSMENT INSTRUMENTS

Currently, the diagnosis of DILI relies primarily on expert opinion and clinical judgment after exclusion of more common causes of liver injury with a compelling temporal association between drug exposure and injury onset and improvement following drug cessation. When the diagnosis of DILI is suspected, it is important to obtain a thorough medication history with an associated timeline of drug exposure and inquire about over-the-counter medication and HDS use. The R ratio at liver injury onset can guide additional serologic and imaging tests to narrow the differential diagnosis (Figure 1).

**FIGURE 1:**
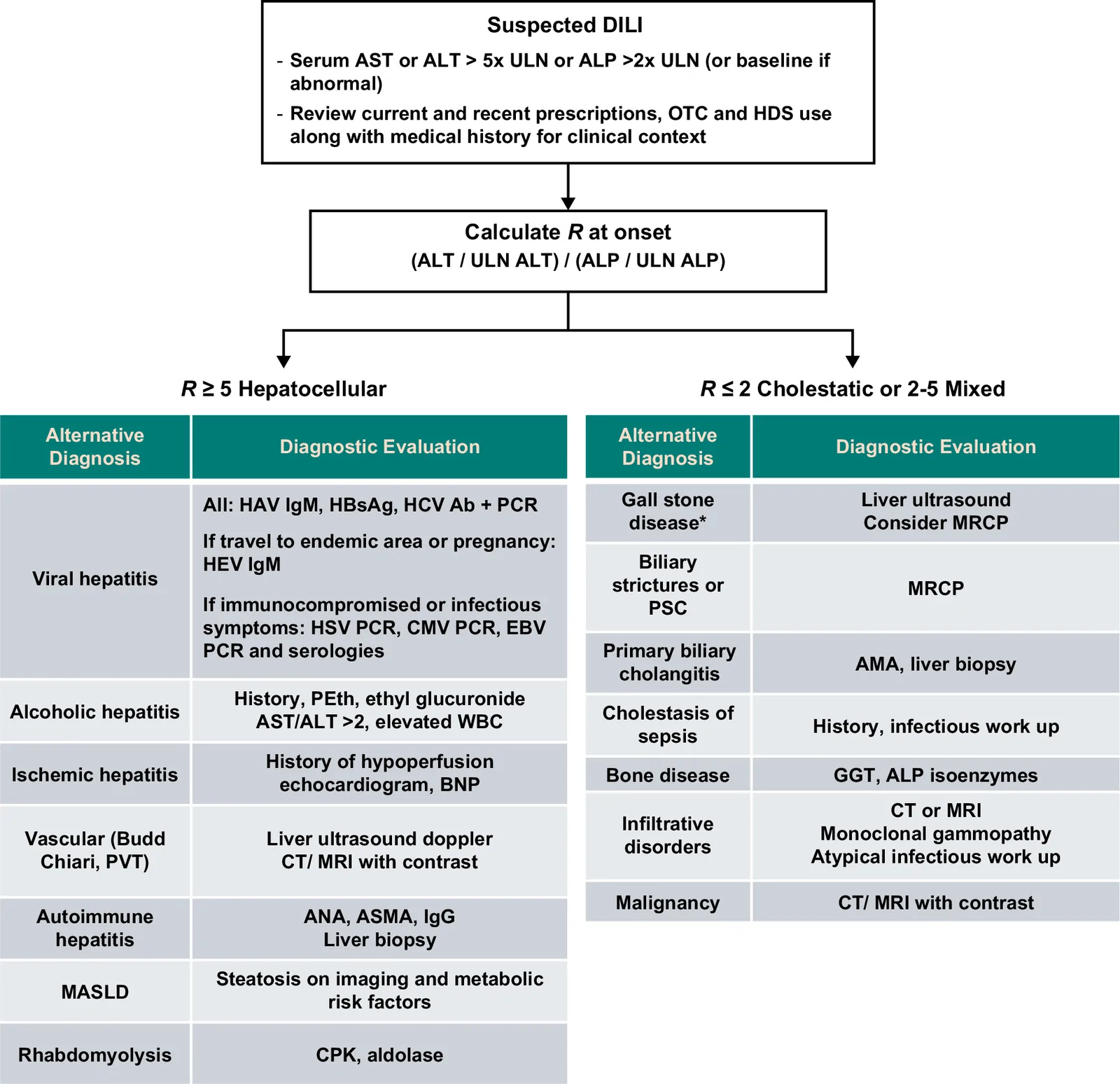
Diagnostic approach to patients with suspected DILI. A detailed history of past and current drug exposures is critical. Calculation of the *R* factor using a patient’s serum ALT and alkaline phosphatase values at liver injury onset can guide further evaluation for competing causes via serologic testing and imaging. Note that acute choledocholithiasis can present with acute hepatocellular injury with an ALT as high as 1000 IU/L in the absence of cholestasis. Abbreviations: Ab, antibody; ALT, alanine aminotransferase; ALP, alkaline phosphatase; AMA, anti-mitochondrial antibody; ANA, antinuclear antibody; ASMA, anti-smooth muscle antibody; AST, aspartate aminotransferase; BNP, B-type natriuretic peptide; CMV, cytomegalovirus; CPK, creatine phosphokinase; CT, computed tomography; EBV, Epstein–Barr virus; GGT, gamma-glutamyl transferase; HAV, hepatitis A virus; HDS, herbal and dietary supplements; HBsAg, hepatitis B surface antigen; HEV, hepatitis E virus; HSV, herpes simplex virus; IgG, immunoglobulin G; IgM, immunoglobulin M; MASLD, metabolic dysfunction–associated steatotic liver disease; MRCP, magnetic resonance cholangiopancreatography; MRI, magnetic resonance imaging; OTC, over-the-counter medications; PCR, polymerase chain reaction; PEth, phosphatidylethanol; PSC, primary sclerosing cholangitis; PVT, portal vein thrombosis; ULN, upper limit of normal; WBC, white blood cell.

Diagnostic testing should be tailored to the individual patient and clinic presentation. All patients should be tested for hepatitis A, B, and C with hepatitis A immunoglobulin M (IgM), hepatitis B surface antigen (HBsAg), hepatitis B core antibody (anti-HBc), and hepatitis C antibody with hepatitis C virus ribonucleic acid (HCV-RNA) if concern for parenteral exposure in the last 12 weeks. Hepatitis B reactivation should be considered when assessing DILI in patients receiving immunosuppressive and oncological therapies. The risk of HBV reactivation with B-cell depleting therapies such as rituximab is widely recognized however recent data have also shown a high risk of reactivation (>10%) in those receiving janus kinase inhibitors and intermediate risk (1%–10%) with cytokine inhibitors (excluding anti-TNF agents), chimeric antigen receptor T-cell immunotherapy, high-dose corticosteroids and calcineurin inhibitors in anti-HBc + patients.^22^ Additional viral testing should be obtained based on patient risk factors and clinical context (Figure 1). Cytomegalovirus should also be considered in immunocompromised patients, including solid organ transplant recipients. Younger adults and teenagers who present with symptoms of mononucleosis, such as fever, malaise, or adenopathy, should be evaluated for Epstein–Barr virus (EBV) infection using a combination of serologies and EBV-DNA testing. History of alcohol use, metabolic risk factors such as obesity, diabetes, and hyperlipidemia, and episodes of hypotension or hypoperfusion can also provide insight into the etiology of abnormal liver tests. The imaging modality used should also be based on the suspected cause of the injury (Figure 1). After an additional workup excludes competing etiologies, standardized causality assessment instruments can help determine the probability of DILI.

The Roussel Uclaf Causality Assessment Method (RUCAM) has been the primary standardized scoring system to assist in determining the likelihood of DILI since 1993.^23^ In 2022, a Revised Electronic Causality Assessment Method (RECAM) was published using prospectively identified single-drug cases from DILIN and the Spanish DILI registry.^24^ In addition to creating a computerized interface with detailed user instructions, the RECAM removed non–evidence-based risk factors (age, alcohol, pregnancy), anchored hepatoxicity scoring to LiverTox likelihood scores, and added an optional domain 5 to include liver histology and rechallenge data when available (Table 2). With these changes, the RECAM had significantly greater sensitivity in identifying the extremes of likelihood categories (definite/highly likely and unlikely/excluded). Although improved and easier to use than the RUCAM, the RECAM still only had 62% agreement with expert opinion.^24^

**TABLE 2:** The RECAM instrument data domains and interpretation

Domain	Points			
1a Drug start to onset	−6 to +4			
1b Drug discontinuation to onset	−6 to 0			
2 Dechallenge	−6 to +4			
3 LiverTox score	0 to +3			
A (>50 cases)	3			
B (12–50 cases)	3			
C (3–12 cases)	1			
D (<3 cases)	1			
E*	1			
E or Xm (0 cases or unknown)	0			
4 Competing causes	−6 to +1			
5 Optional data				
Rechallenge	−3 to +6			
Liver histology	−6 to +1			
CMV testing	−6 to 0			
EBV testing	−6 to 0			
HSV testing	−6 to 0			
DRESS	0 to +1			
RECAM score	−8 to +18			
RECAM score interpretation				
≥8	Highly likely/highly probable			
4–7	Probable			
3 to +3	Possible			
≤4	Unlikely/ excluded			
**Domain**	**Points**	**LiverTox likelihood category**		**RECAM points**
1a Drug start to onset	−6 to +4	A (well-known)	50 cases	3
1b Drug discontinuation to onset	−6 to 0			
2 Dechallenge	−6 to +4	B (known highly likely)	12–50 cases	
3 LiverTox score	0 to +3	C (known probable)	3–12 cases	1
4 Competing causes	−6 to +1	D (rare but possible)	<3 cases	
5 Optional data				
Rechallenge	−3 to +6	E* (unlikely)	Clinical trial or FDA reports only	
Liver histology	−6 to +1			
CMV testing	−6 to 0			
EBV testing	−6 to 0			
HSV testing	−6 to 0			
DRESS	0 to +1			
RECAM score	−8 to +18	E (unlikely)	No cases	0
		X (new med or rarely used)	Unknown	
RECAM score interpretation				
≥8	Highly likely/highly probable			
4–7	Probable			
3 to +3	Possible			
≤−4	Unlikely/excluded			

*Note:* E* = reported in clinical trials or by the FDA alone.

Adapted from Hayashi et al.^24^

Adapted from Hayashi et al.^23^

Abbreviations: CMV, cytomegalovirus; DRESS, drug-related eosinophilic systemic syndrome; EBV, Ebstein–Barr virus; FDA, Food and Drug Administration; HSV, herpes simplex virus; RECAM, Revised Electronic Causality Assessment Method.

Several groups have recently set out to further improve the RECAM instrument. The group from Cincinnati reported 123 suspected DILI cases and found that removal of the requirement for HCV-RNA and HEV testing would improve the scoring system compared with expert opinion.^25^ Similar findings were reported by the Japanese.^26^ Future incorporation of genetic polymorphism data into domain 5 could further improve RECAM performance and drug class-specific RECAM instruments.

Although a liver biopsy is not required to make a diagnosis of DILI, many hospitalized patients with severe DILI and those who do not improve after drug discontinuation undergo a diagnostic liver biopsy. Kleiner et al^27^ demonstrated 6 histological patterns commonly seen in bona fide DILI cases. In addition, the presence of eosinophils and granulomas was associated with a more favorable prognosis, while hepatic fibrosis and ductular reaction were associated with a poorer prognosis. More recently, DILIN demonstrated that the availability of histological data increased the certainty of DILI diagnosis.^28^

## GENETIC TESTING

Several polymorphisms in human leukocyte antigen (HLA) class I and II alleles have been associated with drug-specific idiosyncratic DILI (Table 3).^29^^–^^37^ HLA polymorphisms shown to be significantly associated with multiple drugs include HLA-A*02:01 for AC and cefazolin DILI and HLA-B*35:01 for multiple HDS products, including green tea extract, turmeric, and *Polygonum multiform*. These data suggest that individuals with these polymorphisms may be more susceptible to DILI from multiple drugs and highlight the important role of the adaptive immune response in the development of idiosyncratic DILI through drug-derived antigens triggering antigen-specific T-cell responses in genetically susceptible individuals. In addition to HLA associations, variants in *PTPN22* are associated with an increased risk of DILI from most prescription drugs in all patient ethnic groups.^38^ *PTPN22* encodes a tyrosine phosphatase that dampens T-cell receptor signaling, and polymorphisms have been associated with several autoimmune diseases.^39^ Alongside HLA risk alleles, PTPN22 may influence the magnitude and persistence of the adaptive immune response to drug-specific antigens in predisposed individuals. DILIN reported on a 5-gene polygenic risk score involving variants in HLA-A*02:01, HLA-B*15:18, HLA-DRB1*15:01, PTPN22, and ERAP-2 for patients with AC DILI that outperformed models involving individual variants.^40^

**TABLE 3:** Examples of drug-specific HLA genetic polymorphism associations and DILI susceptibility

Agent	HLA gene	Race/ethnicity	Case MAF	Control MAF	OR (95% CI)
Prescription drugs					
Amoxicillin–clavulanate	HLA-DRB1*1501–DQB1*0602	NHW	0.30	0.14	2.8 (2.1–3.8)
	HLA-A*02:01	NHW	0.49	0.28	2.3 (1.8–2.9)
Cefazolin	HLA-A*02:01	All	0.51	0.21	2.9 (1.9–4.6)
Flucloxacillin	HLA-B*57:03	NHW	0.42	0.04	36.3 (26.1–51.3)
Minocycline	HLA-B*35:02	NHW	0.08	0.002	29.6 (7.8–89.8)
Terbinafine	HLA-A*33:01	NHW	0.5	0.01	40.5 (12.5–131.4)
Trimethoprim–sulfamethoxazole	HLA-B*14:01	NHW	0.049	0.009	9.2 (3.2–22.4)
	HLA-A*34:02	NHW	0.02	0.001	47.5 (5.2–320.4)
	HLA-B*35:01	African American	0.25	0.09	13.5 (2.5–48.0)
Herbal and dietary supplements					
Green tea extract	HLA-B*35:01	NHW	0.42	0.06	6.8 (5–19)
Turmeric	HLA-B*35:01	NHW	0.45	0.06	7
Polygonum multiforme	HLA-B*35:01	Asian	0.45	0.03	30.4 (11.7–77.8)

Abbreviations: CI, confidence interval; DILI, drug-induced liver injury; HLA, human leukocyte antigen; MAF, minor allele frequency; NHW, non-Hispanic Whites; OR, odds ratio.

## NEWLY REPORTED CAUSES OF DILI

### Immune checkpoint inhibitors

The therapeutic landscape in oncology has substantially changed over the last 10 years, shifting away from cytotoxic chemotherapy to more molecularly targeted treatments such as kinase inhibitors (KIs) and immune-based therapies. Immune-mediated liver injury from checkpoint inhibitors (ILICIs) is an increasingly recognized cause of liver injury with an incidence of 5%–10%.^41^^–^^43^ Patients who receive anti-CTLA-4 monoclonal antibody infusions, dual immune checkpoint inhibitor (ICI) therapy, or a combination of ICI with cytotoxic chemotherapy or targeted therapies are at increased risk of ILICI in comparison to monotherapy PD-1/PD-L1 inhibitors.^44^ Patients with hepatocellular carcinoma (HCC) are at the highest risk of ILICI, with an incidence of up to 20%, likely due to their underlying liver disease and higher burden of chronic viral infections.^45^ The pattern of liver injury with ILICI is hepatocellular in 40%–50% of patients, with older individuals more likely to present with a mixed or cholestatic profile, and typically occurs in the first 6 months of therapy.^42^^,^^43^^,^^46^ A diagnosis of ILICI requires exclusion of alternative etiologies via serological testing along with contrast-enhanced cross-sectional imaging of the liver to exclude hepatic metastases and biliary obstruction.^42^^,^^43^ Interestingly, ILICI patients have a better short-term prognosis and survival compared with ICI-treated patients with liver injury attributed to disease progression or other causes.^42^^,^^43^

A variety of histopathologic findings have been reported in ILICI patients, including noncaseating granulomas, lobular hepatitis, pericentral necrosis, hepatocyte apoptosis, and endothelitis, as well as portal-based inflammation in those with cholestatic liver injury.^47^^,^^48^ Although the findings are not pathognomonic for ILICI, they can help rule out alternative etiologies of liver injury such as microscopic metastases.^49^ Clinically, ILICI tends to be mild, with 70%–80% of patients normalizing their liver tests to pretreatment baseline after withholding the drug and administration of corticosteroids. A recent retrospective cohort of nearly 300 non-hepatocellular carcinoma cancer patients who developed ILICI found that the biochemical pattern of liver injury at onset, liver biopsy features, and severity at onset did not influence time to ALT improvement and steroid responsiveness.^50^

### Kinase inhibitors

Host protein kinases have a pivotal role in cell signaling and in the development of cancer, prompting KIs to be extensively investigated as antineoplastic therapies. There are currently 85 FDA-approved KIs for cancer, autoimmune, and inflammatory conditions, which most frequently target EGFR, JAK, VEGFR, BCR-Abl, ALK, and cyclin-dependent kinases.^51^ More than half of the KIs were associated with mild, reversible elevations in serum AST and ALT levels in clinical trials.^52^ Although severe hepatotoxicity with jaundice from KIs develops in <5%, fulminant hepatitis leading to acute liver failure and an autoimmune phenotype has been reported.^52^ In DILIN, the most common KIs associated with liver injury are imatinib, pazopanib, dabrafenib, lapatinib, and ribociclib for breast cancer.^53^ As the number and mechanistic diversity of KIs further expand, awareness of the potential for DILI is important, along with regular laboratory monitoring.

### Immunomodulatory agents

Recent case series have demonstrated clinically significant liver injury in inflammatory bowel disease (IBD) patients receiving anti-tumor necrosis factor (TNF) α agents, with an over-representation of infliximab. The majority of these patients present within 6 months with a severe acute hepatocellular injury and have detectable serum autoantibodies and an autoimmune-like hepatitis on biopsy. Although many of these patients required short-term corticosteroids, some were successfully rechallenged with other anti-TNFα inhibitors.^54^^,^^55^ Interestingly, another case series from DILIN demonstrated that some patients with multiple sclerosis who received high doses of intravenous (i.v.) methylprednisolone (1000–1500 mg) may develop severe acute hepatocellular injury.^56^

### Herbal and dietary supplements

A recent population-based study demonstrated that nearly 15 million Americans are using botanical products for general well-being.^57^ In DILIN, there has been an increase in turmeric-associated DILI cases.^33^ Turmeric and its primary bioactive ingredient, curcumin, are taken for their anti-inflammatory properties and frequently combined with piperine (black pepper) to increase the oral bioavailability in commercial products. In a recent chemical analysis of the products taken by 7 patients with turmeric-associated DILI, 3 contained piperine.^33^ Most patients with turmeric hepatotoxicity were female (80%) and presented with an acute hepatocellular injury profile, 50% were hospitalized, and 1 patient (11%) died. Interestingly, HLA-B*35:01 was associated with turmeric hepatotoxicity, as it had been previously associated with green tea extract hepatotoxicity.^32^ Other HDS products associated with DILI include ashwagandha, used for centuries in traditional Indian medicine, which is now being marketed for anxiety and stress reduction.^58^ The liver injury was cholestatic or mixed in all of the ashwagandha DILI patients, and most of them had protracted jaundice and pruritus, but no fatalities were reported.^58^ Kratom, which acts as an opioid receptor agonist, can also cause hepatotoxicity with similar features of mixed or cholestatic injury and a prolonged course with jaundice and synthetic dysfunction.^59^ Of note, several HDS, including extract from *Silybum marianum* (milk thistle), *Phyllanthus niruri* (Chanca piedra/Gale-of-the-wind/Bhumi amla), *Camellia sinensis* (green tea), and *Coffea arabica* (coffee), are marketed and consumed by patients for liver disease prevention and management despite well-documented cases of DILI due to green tea extract in the literature.^32^^,^^60^ Understanding a patient’s health and cultural motivations for use of HDS and providing patients with the supportive evidence for their use, as well as the documented harms, can assist in shared decision-making between the patient and provider.^61^

### Medications for patients with liver disease

Naltrexone is an opioid receptor antagonist that is used to treat obesity, drug addiction, and alcohol use disorder (AUD). In a case series of hepatotoxicity attributed to AUD medications, disulfiram was most commonly implicated, with only 1 case attributed to naltrexone despite its widespread and increasing use.^62^ Furthermore, another single-center study failed to demonstrate any evidence of DILI in 220 patients with clinically significant alcoholic liver disease treated with acamprosate, naltrexone, or disulfiram.^63^ However, most experts advise against the use of disulfiram in patients with clinically significant liver disease.

Obeticholic acid is a farnesoid X receptor agonist that was approved as a second-line agent for primary biliary cholangitis (PBC) in 2016. Due to cases of potentially severe idiosyncratic DILI, a black-box warning and prescribing restrictions were issued in 2020.^64^ Clinical trials of obeticholic acid in non-cirrhotic metabolic dysfunction–associated steatotic liver disease (MASLD) failed to demonstrate efficacy and were also associated with a higher rate of liver injury compared with placebo, and the drug was recently withdrawn from the market.^65^ Despite their widespread use by millions of patients, there have only been a handful of DILI cases attributed to glucagon-like peptide-1 (GLP-1) receptor agonists, although cholecystitis is more frequent compared with placebo-treated patients.^66^^–^^68^

The “caftors” (ivacaftor, deutivacaftor, lumacaftor, tezacaftor, elexacaftor, vanzacaftor) are orally administered potentiators of the cystic fibrosis transmembrane regulator (CFTR) that were approved in 2012 for patients with specific cystic fibrosis (CF) genotypes that can lead to dramatic improvements in lung function and nutritional status. Combinations of 3 of these modulators, such as ETI or VTD, have become the standard of care for up to 85% of CF patients. During clinical trials, up to 25% of CF patients treated with ivacaftor had a mild transient serum ALT elevation without symptoms that frequently resolved without drug discontinuation.^69^ In post-marketing surveillance studies of triple therapy, case reports of clinically significant liver injury have been reported at several weeks to months of treatment that improved with drug discontinuation. However, instances of liver failure, liver transplantation, and death have also been reported.^70^ Furthermore, acute cholecystitis after initiation of caftor therapy has been reported, presumably due to increased clearance of sludge with enhanced bile flow.^71^ As a result, monthly monitoring of liver biochemistries is recommended for the first 6 months of caftor therapy and then every 3–6 months thereafter. The drug should be held if patients develop symptoms, jaundice, or liver chemistries more than 5 times their baseline.

## TREATMENT OF DILI

The initial recommended treatment of DILI is to withhold the suspect agent(s) and any other non-essential medications and provide supportive care, including fluids, antiemetics, and antipruritics.^72^ A recent systematic review demonstrated a potential role for short-term corticosteroids in highly selected subgroups of DILI patients, including those with ILICI or drug-induced autoimmune hepatitis (DI-AIH), but not in moderate to severe idiosyncratic DILI or severe acute liver injury (ALI) or ALF.^73^ It is important to note that most of the studies included were observational without contemporary controls. In one study, patients with TMP-SMX hepatotoxicity and jaundice or hypersensitivity features receiving short-term corticosteroids experienced more rapid improvement in liver biochemistries compared with propensity-matched controls.^74^ In addition, patients with drug reaction with eosinophilia and systemic symptoms (DRESS) who have significant liver involvement should be treated with high-dose corticosteroids in conjunction with an experienced dermatologist.^75^

### ILICI management

The treatment of ILICI depends on the toxicity grade (Table 4). Although corticosteroids remain the standard treatment for grade 3–4 liver injury, studies have shown that 33%–50% of patients may improve without treatment.^73^ Management of grades 1 and 2 hepatotoxicity includes biweekly lab monitoring and holding the ICI. Persistent grade 2 hepatotoxicity despite drug withdrawal and grade 3 should be treated with corticosteroids, typically oral prednisone 0.5–1 mg/kg with taper by 10 mg weekly. Hospitalization is recommended for a grade 4 injury for initiation of i.v. corticosteroids and clinical monitoring. The optimal dose of steroids for ILICI has not been well elucidated. In a retrospective analysis of patients with high-grade ILICI who received initial doses of either ≥1.5 mg/kg versus <1.5 mg/kg methylprednisolone equivalents, there was no difference in time to normalization of ALT or development of steroid-refractory disease.^76^ However, lower steroid doses were associated with significantly lower infectious complications and hyperglycemia. In ILICI patients who are steroid non-responders, the addition of an anti-metabolite such as azathioprine or mycophenolate can improve liver chemistries in uncontrolled studies.^77^ However, the criteria of when to consider calcineurin inhibitors, anti-TNFα agents, i.v. immunoglobulin, or anti-thymocyte globulin remain unclear due to the small number of patients treated.

**TABLE 4:** Common terminology criteria for adverse events version 5.0 grading system for immune checkpoint inhibitor hepatotoxicity

	Serum AST/ALT	Alk phos	Total bilirubin	Treatment
Grade 1	>ULN–3.0 × ULN or ^a^	>ULN–2.5 × ULN or ^a^	>ULN–1.5 × ULN or ^a^	Hold ICI and biweekly labs
Grade 2	>3.0–5.0 × ULN or ^a^	>2.5–5.0 × ULN or ^a^	>1.5–3.0 × ULN or ^a^	Hold ICI, frequent labs, oral steroids
Grade 3	>5.0–20.0 × ULN or ^a^	>5.0–20.0 × ULN or ^a^	>3.0–10.0 × ULN or ^a^	Discontinue ICI, i.v., or oral steroids, GI consult
Grade 4	>20.0 × ULN or ^a^	>20.0 × ULN or ^a^	>10.0 × ULN or ^a^	Stop ICI, hospitalize, i.v. steroids, GI consult
Grade 5	Death	Death	Death	

^a^ Abnormal baseline.

Abbreviations: Alk phos, alkaline phosphatase; ALT, alanine aminotransferase; AST, aspartate aminotransferase; GI, gastroenterology; ICI, immune checkpoint inhibitor; i.v., intravenous; ULN, upper limit of normal.

### N-acetylcysteine for severe DILI

N-acetylcysteine (NAC) is the standard treatment for all patients with acetaminophen overdose (APAP) and hepatotoxicity, with emerging data regarding its use in non-APAP ALF. In patients with non-APAP ALF with early-stage encephalopathy, a randomized controlled trial found that i.v. NAC for 72 hours improved transplant-free survival in those with DILI from 27% to 58%.^78^ However, there was no overall survival benefit at 3 weeks. A systematic review of 11 studies found similar improvement in transplant-free survival but inconsistent benefit in overall survival.^79^ Of note, NAC is not recommended for children with non-APAP ALI or ALF due to lower 1-year survival.^80^

### Ursodeoxycholic acid

Ursodeoxycholic acid (UDCA) is a synthetic bile acid approved for PBC and is frequently used off-label for patients with severe cholestatic DILI or associated pruritus. Small case series have shown improvement in liver biochemistries with varying doses of UDCA; however, multiple meta-analyses have shown inconsistent benefit of UDCA in the resolution of DILI.^81^^,^^82^ Due to the lack of randomized control trials, the decision about when and how to use UDCA in the treatment of DILI needs to be made on a case-by-case basis. In patients with secondary sclerosing cholangitis from ICI, a trial of UDCA is preferred over corticosteroids, as this phenotype is generally not responsive to escalation of immunosuppression.^83^ Furthermore, a French group has recently demonstrated that UDCA may lead to laboratory improvement in up to 80% of ILICI patients with cholestatic DILI.^84^

### Outcomes and prognosis

Over 80% of DILI patients will fully recover within 6 months of drug discontinuation with supportive care, and only a small proportion of patients (4.6%–17.1%; Table 1) go on to develop chronic DILI at 6 months.^8^ Patients with vanishing bile duct syndrome on biopsy have a poorer long-term outcome and frequently remain symptomatic for many months or years.^85^ A model consisting of the Charlson comorbidity index combined with Model for End-Stage Liver Disease (MELD) scores and serum albumin level predicted 6-month mortality with a high degree of accuracy in a test and validation cohort from DILIN.^86^

Hospitalized patients with DILI-related ALF may require consideration for liver transplantation since less than 50% of patients spontaneously recover.^16^ Fortunately, the proportion of liver transplants due to DILI-related ALF is declining over time, possibly due to increased awareness, improvements in critical care, and/or increased use of NAC.^16^ However, patients with ALF due to HDS have a significantly poorer survival compared with those with DILI from a drug.^16^ Multiple prognostic models have been developed to identify ALF patients with a poor prognosis. A recently published study of 305 inpatients with severe DILI-related ALI or ALF identified 5 variables associated with 21-day transplant-free survival (use of pressors, HE grade, initial bilirubin, initial INR, HDS).^87^ The DILI-Inpt Prognostic score was superior to the MELD and King’s College criteria score in predicting outcomes.^87^ This simple scoring system may prove to be a useful bedside tool if further validated in other cohorts.

## SUMMARY AND FUTURE DIRECTIONS

Substantial challenges remain in establishing a clinical diagnosis of idiosyncratic DILI. Although treatment of DILI largely involves supportive care, emerging data support the use of UDCA in selected patients with cholestatic ILICI and pruritic DILI, corticosteroids in selected patients with moderate to severe autoimmune-like hepatitis that does not improve with drug discontinuation, and NAC for DILI-related ALF (Table 5). The development of the RECAM instrument and LiverTox database with objective and semi-quantitative scales is a major advance in DILI clinical care and research. To address the increasing incidence of HDS hepatotoxicity, a collaboration between DILIN and the FDA to more rapidly identify and confirm new causes of HDS hepatotoxicity was recently initiated. The HDS Sentinel Surveillance System with suspect product ingredient analysis will hopefully lead to earlier identification and regulatory actions regarding potentially hepatotoxic products in the marketplace.^88^

**TABLE 5:** Key clinic pearls in drug-induced liver injury in 2026

DILI diagnosis
- Obtain detailed history of both current and recent use of all prescription medications, vaccines, over-the-counter medications, and herbal/botanicals, including single exposures (periprocedural antibiotics, cancer infusions) in the past 3 mo
- Consider HBV reactivation in patients receiving chemotherapy or immunomodulatory biologics who are HBsAg + or have prior exposure (ie, anti-HBc+)
- When considering acute HCV, EBV, or CMV infection, HCV-RNA, EBV-DNA, and CMV-DNA testing is recommended over serologies
- LiverTox is a widely available authoritative resource with clinical hepatotoxicity data and likelihood scores on over 1200 drugs and 160 HDS products (see LiverTox—NCBI Bookshelf)
- The RECAM is a free, computerized causality assessment instrument that can assist with DILI diagnosis (see DILI RECAM—Causality Assessment)
DILI management
- Withholding the suspect drug and supportive measures leads to DILI resolution in over 80% of cases within 6 mo
- Refer all DILI-related ALF cases to the nearby liver transplant center in light of poor spontaneous survival (30%). Consider N-acetylcysteine infusion for hospitalized DILI-related ALF cases, as well as all acetaminophen overdose cases
- Corticosteroids (1 mg/kg oral or i.v.) are recommended for patients with grade 3 or higher ILICI. Some steroid-refractory ILICI patients may require mycophenolate mofetil or additional therapies
- Corticosteroids are recommended in rare DILI patients with DRESS or severe hypersensitivity features. However, the optimal dose and duration of corticosteroids in selected patients with biopsy-confirmed autoimmune-like features and worsening lab parameters are unknown
- Ursodeoxycholic acid is frequently used in DILI patients with symptomatic pruritus or prolonged cholestasis, but the clinical benefit is uncertain due to a lack of controlled trials

Abbreviations: ALF, acute liver failure; anti-HBc, antibody to hepatitis B core antigen; CMV, cytomegalovirus; CMV-DNA, cytomegalovirus DNA; DILI, drug-induced liver injury; DRESS, drug reaction with eosinophilia and systemic symptoms; EBV, Epstein–Barr virus; EBV-DNA, Epstein–Barr virus DNA; HBsAg, hepatitis B surface antigen; HBV, hepatitis B virus; HCV, hepatitis C virus; HCV-RNA, hepatitis C virus RNA; HDS, herbal and dietary supplement; ILICI, immune-mediated liver injury caused by immune checkpoint inhibitors; i.v., intravenous; RECAM, Revised Electronic Causality Assessment Method.

Multiple lines of evidence suggest that aberrant adaptive host immunity is involved in DILI pathogenesis. Hopefully, further studies using whole-exome sequencing will help identify causal variants involved in DILI susceptibility and outcome. Finally, the development of in vitro test systems using human liver organoids and induced pluripotent stem cells holds great promise for improving DILI diagnosis and pathogenesis studies.^89^ Clearly, much progress has been made in DILI diagnosis and treatment over the past 20 years, but more studies are now needed involving carefully phenotyped patients enrolled in DILI registries to improve our understanding of this complex, multifaceted, and fascinating disease.
